# Effect of Melatonin Gel as an Adjunct to Non-Surgical Periodontal Therapy: A Systematic Review of Randomized Controlled Trials

**DOI:** 10.3390/jcm15072624

**Published:** 2026-03-30

**Authors:** Thaleia Angelopoulou, Yiorgos A. Bobetsis

**Affiliations:** 1 School of Medicine, National and Kapodistrian University of Athens, 11527 Athens, Greece; thangelop@yahoo.gr; 2 Department of Periodontology, School of Dentistry, National and Kapodistrian University of Athens, 11527 Athens, Greece

**Keywords:** melatonin, melatonin gel, periodontitis, non-surgical periodontal therapy, NSPT, adjunctive therapy, local drug delivery, systematic review

## Abstract

**Background/Objectives**: Melatonin, an antioxidant agent with anti-inflammatory and immunomodulatory properties, has recently been investigated as an adjunct to non-surgical periodontal therapy (NSPT). While most studies have focused on systemic administration, a limited number of recent clinical trials have evaluated the local delivery of melatonin in gel form. This systematic review evaluated the clinical and radiographic outcomes of locally delivered melatonin gel as an adjunct to NSPT in patients with periodontitis. **Methods**: Four databases (PubMed, Scopus, Web of Science, Cochrane Library), along with the gray literature, were systematically searched until November 2025. Randomized controlled trials (RCTs) investigating the effect of locally delivered melatonin gel as an adjunct to NSPT on clinical and radiographic periodontal parameters in patients with periodontitis compared with NSPT alone or combined with placebo administration were included. Assessment of risk of bias and certainty of evidence was conducted. **Results**: Five RCTs were eligible for qualitative synthesis. With a low level of certainty, the use of melatonin gel as an adjunct to NSPT was generally associated with significant improvements in clinical periodontal parameters and radiographic outcomes compared with NSPT plus placebo. Meta-analysis was not feasible due to substantial methodological heterogeneity across studies, including differences in study design, reporting, and outcome assessment. **Conclusions**: Adjunctive use of melatonin gel with NSPT may provide additional clinical and radiographic benefits in patients with periodontitis. However, the certainty of evidence is low; therefore, conclusions should be interpreted with caution. Evidence remains limited regarding the optimal melatonin concentration and dosing regimen. Well-designed, adequately powered RCTs with standardized protocols and longer follow-up periods are required to confirm these findings and define optimal use.

## 1. Introduction

Periodontitis is a chronic multifactorial inflammatory disease driven by the dynamic interplay between bacterial biofilm and the host immune response, leading to the progressive destruction of the tooth-supporting tissues [1]. Initial management of periodontitis relies on non-surgical periodontal therapy (NSPT), which aims to reduce the periodontal inflammatory burden and control the etiologic biofilm [2,3,4]. Within contemporary treatment frameworks, NSPT corresponds to Steps 1 and 2 of periodontal therapy and constitutes the first phase of periodontal treatment. Clinical outcomes following NSPT are subsequently evaluated to determine the need for adjunctive approaches and/or periodontal surgery.

Despite the effectiveness of NSPT, limitations in clinical outcomes at specific sites or in specific patient groups have driven interest in adjunctive therapies to enhance treatment response. Accordingly, numerous adjunctive strategies have been explored to improve clinical outcomes, particularly in challenging scenarios, such as non-responsive sites with persistent deep pockets, intrabony defects, and furcation involvement, and in patients with systemic conditions that may compromise periodontal healing [5,6,7,8,9]. Adjunctive agents, including antibiotics, probiotics, host-modulating agents, and antioxidants, may be delivered systemically or locally in conjunction with NSPT [10,11,12]. Evidence suggests that such approaches may support improved outcomes by reducing inflammation, limiting oxidative stress, and attenuating periodontal tissue breakdown [13,14,15,16,17,18].

Among antioxidant agents, melatonin has attracted increasing interest. Melatonin is an endogenous hormone produced by the pineal gland that is responsible for the regulation of circadian rhythms [19]. Importantly, it also exhibits antioxidant, anti-inflammatory, and immunomodulatory properties [20]. In the oral cavity, melatonin is present in saliva and gingival crevicular fluid (GCF), and higher salivary melatonin levels have been associated with better periodontal status [21]. When administered systemically as an oral supplement alongside NSPT, it improves clinical periodontal parameters and oxidative stress biomarkers in both systemically healthy patients [22,23,24] and patients with diabetes [25,26,27,28,29,30,31]. These effects may be explained by melatonin’s biological activities, including stimulation of antioxidant enzyme activity, promotion of collagen synthesis and angiogenesis, induction of osteoblast proliferation and differentiation, and inhibition of osteoclast activity, mechanisms that may support alveolar bone preservation and periodontal regeneration [32,33,34,35].

Despite these potential advantages, systemic melatonin supplementation has limitations, including the need for relatively high doses to achieve effective intra-pocket concentrations, which in turn may increase the risk of adverse interactions and reduce patient compliance [36]. Consequently, local delivery systems, such as orabase cream formulations [37,38,39,40], gels [41,42,43,44,45,46], and melatonin-loaded nanoparticles [47], have been developed to enhance adhesion to periodontal tissues and intra-pocket retention while minimizing systemic exposure [48,49].

Although most clinical studies have focused on systemic melatonin administration, a smaller body of research has investigated local application in gel form, often using heterogeneous concentrations and protocols. The limited number of well-designed RCTs and the lack of standardized dosing regimens underscore the need for a systematic evaluation of the existing evidence. Therefore, the aim of this systematic review is to assess the effect of locally delivered melatonin gel as an adjunct to NSPT on clinical periodontal and radiographic parameters in patients with periodontitis compared with NSPT alone or NSPT plus placebo.

## 2. Materials and Methods

### 2.1. Protocol and Registration

This systematic review was conducted in accordance with the Preferred Reporting Items for Systematic Reviews and Meta-Analyses PRISMA 2020 Checklist (Supplementary Materials File S1) [50]. The study protocol was registered in the International Prospective Register of Systematic Reviews (PROSPERO) database under registration number CRD420251156973.

### 2.2. Study Design

This systematic review addressed the following question: Does melatonin gel improve clinical and radiographic periodontal outcomes when used as an adjunct to NSPT in patients with periodontitis?

### 2.3. Eligibility Criteria

#### 2.3.1. Inclusion Criteria

Eligibility criteria were defined using the PICOS (Population, Intervention, Comparison, Outcomes, and Study design) framework. Randomized controlled trials (RCTs) (S) assessing the effect of locally delivered melatonin gel as an adjunct to NSPT (I) on clinical and/or radiographic periodontal parameters (O) in systemically healthy patients with periodontitis (P) compared with NSPT alone or NSPT plus placebo administration (C) were included in this systematic review. Additional inclusion criteria were clearly described intervention protocols concerning both NSPT and local melatonin gel administration and a minimum follow-up of 3 months. Eligible studies included adult human patients (≥18 years old) diagnosed with periodontitis based on clinical parameters. Only peer-reviewed articles written in or translated into the English language were considered.

#### 2.3.2. Exclusion Criteria

Studies were excluded according to the following criteria: non-randomized study designs (e.g., systematic reviews, non-randomized clinical trials, reports, letters, book chapters, editorials, commentaries, personal opinions, case series, and case reports), multiple reports of the same study, studies involving systemic melatonin administration, studies including patients with systemic comorbidities, studies involving smokers, studies including pregnant or lactating women, studies with unclear intervention protocols, and in vitro or animal studies.

### 2.4. Information Sources and Search Strategy

The literature search was concluded on 25 November 2025. Detailed individual search strategies for each of the following databases were developed: PubMed, Web of Science, Scopus and Cochrane Library. A supplementary search of gray literature sources, including BASE, ProQuest, ResearchGate, and Google Scholar, was also performed (Table S1). Retrieved records were exported to Mendeley Refence Manager (version 2.135.0) for deduplication; remaining duplicates were removed manually.

### 2.5. Study Selection

**Figure 1:**
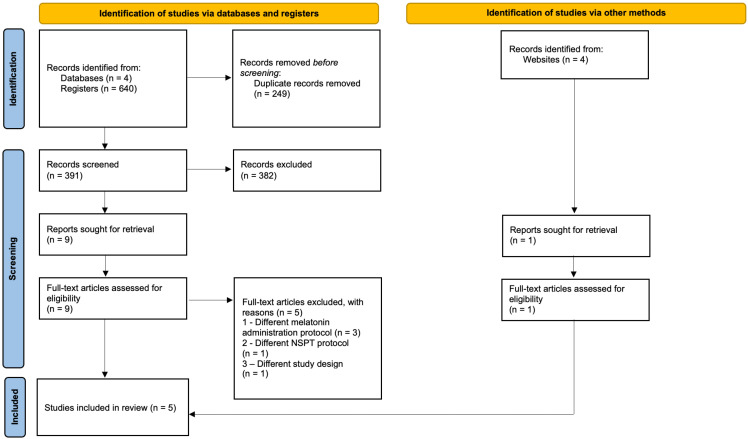
PRISMA flow diagram of the study selection process.

Study selection comprised two phases. Titles and abstracts were independently screened by both reviewers, followed by full-text evaluation of potentially eligible reports. One author (T.A.) further examined the reference lists of all included trials to identify additional eligible studies. Any disagreements regarding study selection were discussed between the two reviewers until consensus was reached. Inter-reviewer agreement during screening was evaluated using Cohen’s kappa coefficient [51]. The study selection process adhered to PRISMA guidelines, as depicted in the flow diagram (Figure 1).

### 2.6. Data Collection and Data Items

Data extraction was independently performed by the two reviewers using a standardized Excel spreadsheet. Information retrieved from the included studies comprised publication details (authors, country, year of publication, journal, language), study design, sample size and participant data (including demographics and periodontitis definition), details of NSPT and melatonin protocols (concentration, delivery method, frequency, duration), follow-up duration, and outcome measures.

### 2.7. Risk of Bias Within Studies

The risk of bias was evaluated independently by the two reviewers using the Risk of Bias in Randomized Studies of Interventions (ROB 2) tool [52] across five domains, leading to an overall judgment of “low risk of bias”, “some concerns”, or “high risk of bias”.

### 2.8. Summary Measures

Clinical periodontal outcomes included changes in probing pocket depth (PPD), clinical attachment level (CAL), and indices regarding gingival inflammation and plaque accumulation. Radiographic outcomes included bone fill of intrabony defects following NSPT.

### 2.9. Certainty of the Evidence

Certainty of evidence was evaluated using the GRADE approach. A Summary of Findings (SoF) table was generated using GRADE-pro software (https://www.gradepro.org, accessed on 10 October 2025). The overall certainty of evidence was classified into four categories (high, moderate, low, very low), taking into consideration risk of bias, inconsistency, indirectness, imprecision, and publication bias, along with additional factors such as the magnitude of effect, the influence of potential confounding, and the presence of dose–response patterns.

## 3. Results

### 3.1. Study Selection

Overall, 640 studies were identified from the electronic databases, with two additional records identified by searching the gray literature. After duplicate removal and screening of titles/abstracts, ten studies underwent full-text assessment. In this second phase, five studies were excluded due to differences in melatonin administration protocols (n = 3), NSPT protocol (n = 1), or study design (n = 1) (Table S2). Therefore, five RCTs were included in the qualitive synthesis. Inter-reviewer agreement was excellent (Kappa > 0.90). No additional eligible studies were identified from the reference lists.

### 3.2. Study Characteristics

All included studies were RCTs published between 2021 and 2025 in English. One study was conducted in Iraq [44], one in Egypt [41], and three in India [42,43,46]. Three trials used a prospective double-blind split-mouth design [42,43,46], one used a prospective triple-blind three-arm design [44], and one used a prospective double-blind three-arm design [41]. A total of 178 subjects, ranging in age from 18 to 65 years old, participated in the studies, and the number of volunteers ranged from 22 to 88 across trials. All studies included patients with periodontitis; three studies [41,42,46] enrolled patients with stage II periodontitis, one [43] study enrolled patients with stage III periodontitis combined with the presence of at least one pair of intrabony defects in either the maxillary or mandibular arch, and one [44] study enrolled patients with both stage II and stage III periodontitis. All studies included systemically healthy participants and excluded smokers, as well as pregnant or lactating women.

All five studies included a melatonin group (melatonin gel as adjunct to NSPT) and a control group receiving NSPT plus placebo gel. Two RCTs included an additional active comparator arm: Vitamin C solution in Rauf et al. [44] and atorvastatin gel in Moussa et al. [41]). These arms were not considered in the present review. In all studies, melatonin and placebo gels were applied in the periodontal pockets once weekly for 4 weeks after NSPT. Melatonin concentrations varied across trials; Pratap et al. [46] and Gonde et al. [43] used 1% melatonin gel, Moussa et al. [41] used 2% gel, and Rauf et al. [44] and Ahmed et al. [42] used 5% gel. In all trials, NSPT consisted of scaling and root planing (SRP) and oral hygiene instructions (OHI); coronal polishing was also performed in one trial [44], and occlusal adjustment was performed when indicated in another trial [43]. Follow-up ranged from 3 to 6 months. An overview of the included studies is presented in Table 1. Due to heterogeneity in methodology and reporting, meta-analysis was not feasible.

### 3.3. Risk of Bias Within Studies

**Figure 2:**
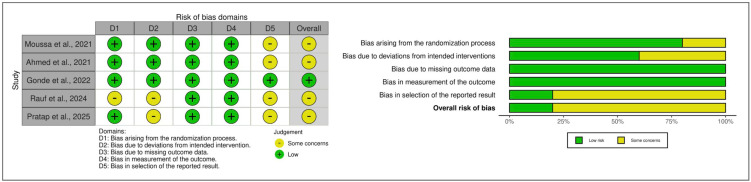
Risk of bias assessment of the included RCTs were assessed using the revised Cochrane Risk of Bias tool for randomized trials (ROB 2; Version 2019) [52] and visualized using the robvis tool [53]. **Left panel**: Risk of bias summary—authors’ assessments of each bias domain for every included study with respect to clinical outcomes. **Right panel**: Risk of bias graph—authors’ judgments about each bias domain presented as percentages across all included studies with respect to clinical outcomes [41,42,43,44,46].

The risk of bias of the five RCTs is summarized in Figure 2. Four trials [41,42,44,46] were indicate to warrant “some concerns”, primarily due to limited information on preregistered study protocols and missing outcome data. One study [43] was judged as having a “low risk” of bias across all domains.

### 3.4. Synthesis of Results and Assessment of the Certainty of Evidence

#### 3.4.1. Changes in Clinical Periodontal Parameters

All included trials evaluated the effect of the adjunctive use of a melatonin gel on periodontal clinical measurements such as PPD and CAL, whereas various periodontal indices were used across studies to assess plaque accumulation and gingival inflammation, including PI, GI, GBI, and mSBI. Overall, both the test and control groups typically improved after NSPT, with melatonin-treated sites/groups often demonstrating greater improvements; however, not all trials reported statistically significant inter-group differences.

Moussa et al. [41], examining 24 participants, reported significant reductions in PPD, CAL, PI, and GI at 3 months in both groups (*p* < 0.0001), with significantly greater improvements in the melatonin group. Ahmed et al. [42], with a total of 24 participants, observed significant reductions in all clinical parameters at 3 months (*p* < 0.001), with greater improvements in PPD and CAL at melatonin-treated sites (*p* < 0.001); inter-group differences for PI and GI were not statistically significant. In the split-mouth study of Gonde et al. [43], comprising 44 patients, statistically significant reductions in PPD, CAL, PI, and mSBI were reported in both groups (*p* < 0.0001), with significantly greater improvements at melatonin-treated sites at 3 and 6 months. In the study of Rauf et al. [44], where 58 patients from melatonin and placebo gel groups were analyzed, a significant reduction in PPD and CAL was observed in both the placebo and the melatonin group from baseline to 3 months (*p* < 0.001). GI also decreased in all groups; however, statistical significance was recorded only at 1 week following NSPT. Overall, the melatonin group exhibited a significantly greater improvement in PPD, CAL, and GI at 3 months. In the split-mouth trial of Pratap et al. [46], including 22 participants, the use of the melatonin gel in conjunction with NSPT led to significant improvements in all evaluated clinical parameters (PPD, CAL, PI, GI, and GBI) from baseline to 3 months (*p* < 0.05). Detailed numerical outcome data are provided in Table S3. The overall certainty of evidence for clinical outcomes was judged to be low (Table S4).

#### 3.4.2. Changes in Radiographic Parameters

Only one trial assessed radiographic outcomes. In the split-mouth study of Gonde et al. [43], where 44 patients were recruited, bone fill and bone volume were evaluated in intrabony defects 6 months post-treatment using cone beam computed tomography (CBCT). Bone fill was defined as the difference in the distance from the cement–enamel junction (CEJ) to the base of the bone defect (BD), while bone volume was calculated by measuring the depth, height, and width of the defects on the CBCT. Both groups exhibited significant intragroup improvements in bone fill and bone volume from baseline to 6 months (*p* < 0.0001). Data from the intergroup analysis demonstrated significantly greater improvements in the 1% melatonin group compared with the placebo group (*p* < 0.0001 for bone fill; *p* < 0.001 for bone volume). More specifically, in the melatonin group, the distance between CEJ and BD decreased by 1.46 ± 0.58 mm, while in the control group it decreased by 0.50 ± 0.38 mm. In a similar trend, the mean bone volume gain of intrabony defects was 51.84 ± 30.23 mm^3^ in the melatonin group and 21.46 ± 8.90 mm^3^ in the placebo group. The level of certainty of evidence for radiographic outcomes was considered to be low (Table S4).

## 4. Discussion

### 4.1. Summary of Evidence

This systematic review investigated whether locally delivered melatonin gel provides additional clinical and radiographic benefits when used as an adjunct to NSPT in systemically healthy patients with periodontitis. Five RCTs, with a total of 172 participants, were included. All studies assessed clinical outcomes, and one of them also analyzed radiographic outcomes. Across studies, adjunctive melatonin gel was consistently associated with significant improvements in clinical periodontal outcomes from baseline to the end of follow-up within each group and, in most trials, with greater improvements compared with the control groups. In the single CBCT-based study assessing radiographic outcomes, adjunctive melatonin was associated with greater improvements in bone fill and bone volume compared with placebo.

Similar findings have been reported in other clinical trials that were not included in this review because they did not meet the eligibility criteria defined for this systematic review. More specifically, in the clinical trial of Tang et al. [45], 1% melatonin gel was used as an adjunct to NSPT in periodontal bone defects in patients diagnosed with stage I and stage IV periodontitis. Compared with NSPT alone, melatonin administration showed significant inter-group improvements in PPD and BI, as well as in bone defect height and alveolar bone density gain; however, follow-up duration was not specified. The authors attributed these clinical and radiographic findings to melatonin’s osteogenic, antioxidant, and anti-inflammatory potential, as reflected by the reductions in IL-1β, IL-6, and TNF-α. Similarly, Al-Agooz et al. [47] reported significant improvements in clinical outcomes and radiographic defect parameters with melatonin-loaded nanoparticles compared with placebo.

Local delivery approaches using melatonin-based orabase formulations have also been investigated in recent years. Evidence suggests that topical 1% melatonin orabase cream applied daily for 20 days, without NSPT, may reduce periodontal inflammation and improve clinical parameters in diabetic patients. These changes were potentially associated with modulation of inflammatory pathways and mediators linked to alveolar bone resorption [37,38,39,40].

The findings of this systematic review align with the results of other reviews on the systemic administration of oral melatonin tablets in both systemically healthy patients [22,23,24] and patients with diabetes [25,26,27,28,29,30,31], highlighting the potential role of melatonin supplementation in NSPT protocols. The observed benefits of melatonin administration in periodontitis management are biologically plausible given melatonin’s antioxidant, anti-inflammatory, and immunomodulatory properties. Melatonin may support periodontal healing following NSPT by enhancing collagen synthesis and angiogenesis and by suppressing pro-inflammatory cytokine activity [25,26,29,30,32,33,34,35]. Moreover, melatonin also induces osteoblast proliferation and differentiation while simultaneously inhibiting osteoclast activity, thereby promoting bone remodeling and mineralization [30,33,34]. These findings could also explain the reported inverse association between GCF and salivary melatonin levels and periodontitis severity [54,55,56].

In this context, melatonin has been explored as a host-modulating agent used adjunctively to NSPT through local delivery in gel form, with the aim of overcoming limitations associated with systemic administration, such as the need for relatively high doses to achieve adequate intra-pocket concentrations, the increased likelihood of systemic interactions, and reduced patient adherence. Although, to the best of our knowledge, no prior systematic review has specifically evaluated melatonin gel as an adjunct to NSPT in the treatment of periodontitis, current evidence suggests that this approach may provide additional benefits in challenging clinical scenarios, such as non-responsive sites with persistent deep pockets, intrabony defects, or furcation involvement, where NSPT alone may not be sufficient.

### 4.2. Limitations and Strengths

Integrating melatonin into NSPT protocols as an adjunct treatment is a relatively new therapeutic concept, and consequently, its application has yet to be implemented in a large number of clinical trials. Hence, following a comprehensive search of electronic databases, the present review ultimately included only five studies, each characterized by a relatively small sample size, limiting the strength and generalizability of the findings. Four studies were judged as warranting some concerns regarding risk of bias, mainly due to insufficient information regarding preregistered study protocols and missing outcome data, whereas a single study demonstrated a low risk of bias. Overall certainty of evidence across outcomes was low, limiting confidence in the conclusions.

A major source of heterogeneity was the varying melatonin concentrations used (1%, 2%, or 5%) across trials, combined with inconsistencies in reporting standards. Moreover, different clinical thresholds were used to define periodontitis, which may have influenced comparability and external validity. Finally, follow-up duration was relatively short and varied across studies (3–6 months), limiting inference regarding long-term stability, delayed effects, and sustained radiography change. Given these sources of heterogeneity, quantitative analysis to determine which concentration produces more favorable clinical or radiographic outcomes was not feasible.

Strengths of this systematic review include a comprehensive search of four major scientific databases and the gray literature. Moreover, study selection, data extraction, and risk of bias evaluation were conducted independently by two reviewers, with any discrepancies resolved through discussion leading to consensus. Additional methodological strengths of this systematic review are the use of validated tools to assess both risk of bias and certainty of evidence across all included studies, along with a detailed evaluation of clinical and radiographic periodontal parameters across all trials, enabling an in-depth synthesis of the available evidence. Furthermore, studies included in this review were exclusively RCTs, originally structured to evaluate a comprehensive range of clinical and, where applicable, radiographic outcomes relevant to our PICO framework. This ensured that both intra- and inter-group treatment responses could be assessed using methodologically appropriate comparisons.

## 5. Conclusions

Melatonin has been investigated as an adjunct to NSPT due to its antioxidant anti-inflammatory and immunomodulatory properties. Local delivery of melatonin aims to increase intra-pocket drug availability while minimizing systemic exposure. Within the limitations of the available evidence, adjunctive melatonin gel may provide additional improvements in key clinical periodontal parameters and radiographic outcomes compared with control therapy. However, these findings are based on a small number of clinical trials and, given the low certainty of evidence presented in this review, current findings should be interpreted with caution.

Given the limited evidence available, well-designed, adequately powered RCTs with standardized NSPT and melatonin protocols, longer follow-up periods, and consistent outcome reporting are needed to confirm these results. Future research should be directed toward defining the optimal melatonin concentration, dosing frequency, and duration of application and should also evaluate the long-term clinical stability of reported outcomes while further exploring its potential effects on radiographic outcomes.

## Figures and Tables

**Table 1:** Summary of descriptive characteristics of included studies.

Author/Year/Country	Study Design	Study Population, Intervention and Control Groups, n	Age Range (years)	Periodontitis Definition	Intervention	Control	Periodontal Parameters Evaluated	Follow-Up (months)	Main Outcomes
Moussa et al., 2021 [41] Egypt	Prospective double-blind three-arm RCT	36 participants: melatonin group, 12 control group, 12	21–55	Stage II Periodontitis [1]	NSPT (OHI, SRP) + Melatonin (2% melatonin gel, intra-pocket application, once weekly for 4 weeks)	NSPT (OHI, SRP) + placebo gel	PPD, CAL, PI, GI	3	Local application of 2% melatonin gel for 4 weeks as an adjunct to NSPT resulted in a significantly greater reduction in PPD, CAL, PI, and GI compared with NSPT plus placebo 3 months post-treatment.
Ahmed et al., 2021 [42] India	Prospective double-blind split-mouth RCT	24 participants	32–55	Stage II Periodontitis [1]	NSPT (OHI, SRP) + Melatonin (5% melatonin gel, intra-pocket application, once weekly for 4 weeks)	NSPT (OHI, SRP) + placebo gel	PPD, CAL, PI, GI	6	Local application of 1% melatonin gel for 4 weeks as an adjunct to NSPT was more effective in improving clinical periodontal parameters in patients with periodontitis compared to placebo.
Gonde et al., 2022 [43] India	Prospective double-blind split-mouth RCT	44 participants	>18	Stage III Periodontitis [1] with intra-bony defects	NSPT (OHI, SRP, occlusal adjustments wherever required) + Melatonin (1% melatonin gel, intra-pocket application, once weekly for 4 weeks)	NSPT (OHI, SRP, occlusal adjustments wherever required) + placebo gel	PPD, CAL, PI, mSBI, Bone fill, Bone volume	3 and 6	Local application of 1% melatonin gel for 4 weeks as an adjunct to NSPT resulted in significant improvements in PPD, CAL, PI, mSBI and radiographic bone fill from baseline to 6 months compared with NSPT plus placebo.
Rauf et al., 2024 [44] Iraq	Prospective triple-blind three-arm RCT	88 participants: melatonin group, 28 control group, 30	18–65	Stage II and III Periodontitis [1]	NSPT (OHI, SRP, coronal polishing) + Melatonin (5% melatonin gel, intra-pocket application, once weekly for 4 weeks)	NSPT (OHI, SRP, coronal polishing) + placebo gel	GI, PPD, CAL	0.25, 1 and 3	Local application of 5% melatonin gel for 4 weeks as an adjunct to NSPT resulted in statistically significant reductions in PPD, CAL and GI from baseline to 3 months in both groups, with the melatonin group showing greater improvement in clinical periodontal parameters compared to NSPT plus placebo.
Pratap et al., 2025 [46] India	Prospective double-blind split-mouth RCT	22 participants	30–45	Stage II Periodontitis [1]	NSPT (OHI, SRP) + Melatonin (1% melatonin gel, intra-pocket application, once weekly for 4 weeks)	NSPT (OHI, SRP) + Placebo gel	PI, GI, GBI, PPD, CAL	1 and 3	Local application of 1% melatonin gel for 4 weeks as an adjunct to NSPT resulted in significant improvements in PPD, CAL, PI, GI and GBI from baseline to 3 months in both the test and the control groups; however, no inter-group differences were statistically significant.

RCT, randomized controlled clinical trial; NSPT, non-surgical periodontal therapy; SRP, scaling and root planing; OHI, oral hygiene instructions; PPD, probing pocket depth; CAL, clinical attachment level; PI, plaque index; GI, gingival index; GBI, gingival bleeding index; mSBI, modified sulcus bleeding index.

## Data Availability

No new data were created or analyzed in this study. Data sharing is not applicable to this article.

## Supplementary Materials

The following supporting information can be downloaded at https://www.mdpi.com/article/10.3390/jcm15072624/s1, Table S1: Detailed search strategy for electronic databases. Table S2: Excluded articles and reasons for exclusion. Table S3: Detailed numerical data for periodontal parameters. Table S4: Quality assessment of included studies; File S1: PRISMA 2020 Checklist.

## Abbreviations

The following abbreviations are used in this manuscript:

NSPT	Non-surgical periodontal therapy
GCF	Gingival crevicular fluid
RCT	Randomized controlled clinical trial
PPD	Probing pocket depth
CAL	Clinical attachment level
PI	Plaque index
GI	Gingival index
GBI	Gingival Bleeding Index
mSBI	Modified sulcus bleeding index
CBCT	Cone beam computed tomography
SRP	Scaling and root planing
CEJ	Cement–enamel junction
BD	Bone defect
OHI	Oral hygiene instruction
LNPs	Loaded nanoparticles
OPG	Osteoprotegerin
RANKL	Receptor Activator of Nuclear Factor-κB Ligand
