# Supplementary material for: Effect of Melatonin Gel as an Adjunct to Non-Surgical Periodontal Therapy: A Systematic Review of Randomized Controlled Trials
Source: J Clin Med. 2026 Mar 30;15(7):2624. doi: 10.3390/jcm15072624 (PMC13072883; doi:10.3390/jcm15072624)
Supplement: Supplementary file 1 [file jcm-15-02624-s001.zip › jcm-4187159-supplementary.pdf]

PRISMA

PRISMA 2020 Checklist

Effect of Melatonin Gel as an Adjunct to Non-Surgical Periodontal Therapy: A Systematic Review of Randomized Controlled Trials

Thaleia Angelopoulou [1] and Yiorgos A. Bobetsis [2,*]

[1] School of Medicine, National and Kapodistrian University of Athens, 11527 Athens, Greece; thangelop@yahoo.gr
[2] Department of Periodontology, School of Dentistry, National and Kapodistrian University of Athens, 11527 Athens, Greece
* Correspondence: ybobetsi@dent.uoa.gr; Tel.: +30-6936613292

Supplementary Materials

Table S1. Detailed search strategy for electronic databases.

Electronic databases

Database	Search (November 25, 2025)	Studies
**PubMed**	("Periodontitis"[MeSH] OR "Periodontal Diseases"[MeSH] OR "Dental Scaling"[MeSH] OR "Debridement"[MeSH] OR "non-surgical periodontal therapy"[tiab] OR "periodontal therapy"[tiab] OR "periodontal treatment"[tiab] OR "periodontitis"[tiab] OR SRP[tiab] OR PDT[tiab]) AND ("Melatonin"[Mesh] OR "melatonin"[tiab] OR "melatonin supplementation"[tiab] OR "melatonin therapy"[tiab] OR "melatonin gel"[tiab])	125
**Web of Science**	(periodontitis OR periodontal disease OR periodontal therapy OR scaling and root planing OR SRP OR PDT) AND (melatonin OR melatonin supplementation OR melatonin therapy OR melatonin gel)	208
**Scopus**	TITLE-ABS-KEY ("periodontitis" OR "periodontal disease" OR "periodontal diseases" OR "non-surgical periodontal therapy" OR "periodontal therapy" OR "periodontal treatment" OR "scaling	233

	and root planing" OR "dental scaling" OR "debridement" OR SRP OR PDT) AND TITLE-ABS-KEY ("melatonin" OR "melatonin supplementation" OR "melatonin therapy" OR "melatonin gel")	
**Cochrane Library**	(non-surgical periodontal therapy OR periodontal therapy OR periodontal treatment OR scaling and root planing OR dental scaling OR debridement OR SRP OR PDT) AND (melatonin OR melatonin supplementation OR melatonin therapy OR melatonin gel)	74

Grey Literature

**BASE**	("periodontitis" OR "periodontal diseases" OR "non-surgical periodontal therapy" OR "periodontal therapy" OR "periodontal treatment" OR "debridement" OR "scaling and root planing" OR SRP OR PDT OR "dental scaling") AND ("melatonin" OR "melatonin supplementation" OR "melatonin therapy" OR "melatonin gel")	528
**ProQuest**	NOFT(("periodontitis" OR "periodontal diseases" OR "non-surgical periodontal therapy" OR "periodontal therapy" OR "periodontal treatment" OR "debridement" OR "scaling and root planing" OR SRP OR PDT OR "dental scaling") AND ("melatonin" OR "melatonin supplementation" OR "melatonin therapy" OR "melatonin gel"))	69
**Google Scholar**	("periodontitis" OR "periodontal diseases" OR "non-surgical periodontal therapy" OR "periodontal therapy" OR "periodontal treatment" OR "debridement" OR "scaling and root planing" OR SRP OR PDT OR "dental scaling") AND ("melatonin" OR "melatonin supplementation" OR "melatonin therapy" OR " melatonin gel")	100
**Research Gate**	"non-surgical periodontal treatment" "melatonin gel"	100

Table S2. Excluded articles and reasons for exclusion.

Records identified from Electronic Databases	
Full-text articles excluded, with reasons (n=5)	
Author/Year	Reason for exclusion
Al-Agooz et al., 2025	1
Dhande et al., 2024	2
Tang et al., 2024	3
Chitsazi et al., 2017	1
Tinto et al., 2020	1

Reasons for exclusion:

1 - Different melatonin administration protocol (n=3)

2 - Different NSPT protocol (n=1)

3 - Different study design (n=1)

Table S3 Detailed numerical data for all periodontal parameters

Periodontal Parameters								
Author/ Year	Group	Follow-up	PPD, mm	CAL, mm	PI, 0-3	GI, 0-3	GBI, % of sites	mSBI, 0-3
Moussa et al., 2021	Control	Baseline	4.66±0.5	4.32±1.0	2.47±0.6	2.62±0.4	-	-
		3 months	3.73±0.5	3.53±0.8	0.91±0.3	1.29±0.4	-	-
	Test	Baseline	4.60±0.6	4.03±0.93	2.08±0.5	2.35±0.5	-	-
		3 months	2.72±0.5	2.28±0.6	0.37±0.1	0.68±0.2	-	-
Ahmed et al., 2021	Control	Baseline	4.0±0.6	4.3±0.6	1.96±0.6	2.4±0.5	-	-
		3 months	3.1±0.7	3.7±0.6	0.7±0.6	0.7±0.5	-	-
	Test	Baseline	4.3±0.8	4.7±0.9	2±0.7	2.4±0.5	-	-
		3 months	2.9±0.7	3.5±0.6	0.7±0.6	0.7±0.5	-	-
Gonde et al., 2022	Control	Baseline – 3 months	-1.50±0.80	+1.68±0.64	-0.75±0.01	-	-	-1.32±0.5
		Baseline – 6 months	-2.40±1.09	+2.72±1.07	-1.18±0.08	-	-	-1.64±0.5
	Test	Baseline – 3 months	-2.09±0.43	+2.29±0.69	-0.75±0.01	-	-	-1.32±0.5

		Baseline – 6 months	-3.50±0.91	+3.79±0.90	-1.18±0.08	-	-	-1.64±0.5
Rauf et al., 2024	Control	Baseline	2.89	1.98	-	2.08	-	-
		1 week	2.88	1.98	-	1.89	-	-
		1 month	2.20	1.95	-	1.29	-	-
		3 months	1.82	1.79	-	0.79	-	-
	Test	Baseline	3.37	2.08	-	2.44	-	-
		1 week	3.29	2.08	-	2.18	-	-
		1 month	2.41	1.91	-	1.26	-	-
		3 months	1.79	1.27	-	0.69	-	-
Pratap et al., 2025	Control	Baseline	5.95±0.78	6.72±1.51	2.17±0.49	2.00±0.00	66.81±15.77	-
		1 month	5.13±0.71	5.77±1.06	2.01±0.36	1.50±0.44	62.50±11.72	
		3 months	4.59±0.79	4.14±0.94	0.81±0.49	0.62±0.36	29.63±15.85	-
	Test	Baseline	5.81±0.73	7.09±1.30	2.02±0.40	2.00±0.00	73.86±12.99	-
		1 month	5.40±0.59	6.18±1.0	1.98±0.34	1.41±0.33	60.50±10.12	
		3 months	4.63±0.79	4.00±0.72	1.00±0.44	0.74±0.35	27.00±13.83	-

Table S4 Quality assessment of included studies

Outcome and follow-up	Patients (studies), N	Certainty	What happens
Change in Probing Pocket Depth (PPD): Follow up: up to 6 months	172 (5 RCTs)	⊕⊕◯◯ Low [a,d]	Statistically significant intragroup PPD reductions were observed in all five RCTs after NSPT. Most trials demonstrated statistically significant greater PPD improvement with adjunctive melatonin compared with control, whereas Pratap 2025 found no significant intergroup difference.
Clinical Attachment Level (CAL): Follow up: up to 6 months	172 (5 RCTs)	⊕⊕◯◯ Low [a,d]	Statistically significant intragroup CAL improvements were reported in all five RCTs following NSPT. Intergroup comparisons generally favored adjunctive melatonin, with larger CAL improvement in melatonin groups in most trials, whereas Pratap 2025 did not demonstrate a statistically significant intergroup difference.
Plaque Index (PI): Follow-up: up to 6 months	114 (4 RCTs)	⊕⊕◯◯ Low [a,d]	Adjunctive application of melatonin gel with NSPT resulted in significant intergroup reductions in plaque index across all trials. Some trials showed a statistically greater reduction in PI in the melatonin-treated group, while others reported no significant intergroup differences.
Gingival Index (GI): Follow up: up to 3 months	128 (4 RCTs)	⊕⊕◯◯ Low [a,d]	Statistically significant intragroup GI reductions were observed in all RCTs following NSPT. Most trials reported greater improvement at melatonin-treated sites, although intergroup differences were not consistently significant across studies.

Outcome and follow-up	Patients (studies), N	Certainty	What happens
Gingival Bleeding Index (GBI): Follow up: 3 months	22 (1 RCT)	⊕⊕◯◯ Low [a,d]	Significant intragroup reductions in GBI were reported in the trial of Pratap 2025 3 months post-treatment in both groups. However, no statistically significant difference was reported between melatonin-treated and control sites.
Modified Sulcus Bleeding Index (mSBI): Follow-up: up to 6 months	44 (1 RCT)	⊕⊕◯◯ Low [d]	Significant intragroup reductions in mSBI were reported in the Gonde 2022 split-mouth trial, with greater improvement at melatonin-treated sites compared with placebo.
Bone Fill: Follow-up: up to 6 months	44 (1 RCT)	⊕⊕◯◯ Low [d]	Significant intragroup improvements in bone fill were observed in the Gonde 2022 split-mouth trial, and melatonin-treated sites demonstrated significantly greater radiographic improvements than the placebo group.
Bone Volume: Follow-up: up to 6 months	44 (1 RCT)	⊕⊕◯◯ Low [d]	Significant intragroup improvements in bone volume were observed in the Gonde 2022 split-mouth trial, and melatonin-treated sites demonstrated significantly greater radiographic improvements than the placebo group.

Reasons for downgrading: a. risk of bias, b. inconsistency, c. indirectness, d. imprecision, e. publication bias

Supplementary Materials File S1

PRISMA 2020 Checklist

Section and Topic	Item #	Checklist item	Location where item is reported
**TITLE**			
Title	1	Identify the report as a systematic review.	1
**ABSTRACT**			
Abstract	2	See the PRISMA 2020 for Abstracts checklist.	1
**INTRODUCTION**			
Rationale	3	Describe the rationale for the review in the context of existing knowledge.	1, 2
Objectives	4	Provide an explicit statement of the objective(s) or question(s) the review addresses.	2
**METHODS**			
Eligibility criteria	5	Specify the inclusion and exclusion criteria for the review and how studies were grouped for the syntheses.	3
Information sources	6	Specify all databases, registers, websites, organisations, reference lists and other sources searched or consulted to identify studies. Specify the date when each source was last searched or consulted.	3
Search strategy	7	Present the full search strategies for all databases, registers and websites, including any filters and limits used.	Table S1
Selection process	8	Specify the methods used to decide whether a study met the inclusion criteria of the review, including how many reviewers screened each record and each report retrieved, whether they worked independently, and if applicable, details of automation tools used in the process.	Table S2
Data collection process	9	Specify the methods used to collect data from reports, including how many reviewers collected data from each report, whether they worked independently, any processes for obtaining or confirming data from study investigators, and if applicable, details of automation tools used in the process.	4
Data items	10a	List and define all outcomes for which data were sought. Specify whether all results that were compatible with each outcome domain in each study were sought (e.g. for all measures, time points, analyses), and if not, the methods used to decide which results to collect.	4
	10b	List and define all other variables for which data were sought (e.g. participant and intervention characteristics, funding sources). Describe any assumptions made about any missing or unclear information.	4
Study risk of bias assessment	11	Specify the methods used to assess risk of bias in the included studies, including details of the tool(s) used, how many reviewers assessed each study and whether they worked independently, and if applicable, details of automation tools	4

Section and Topic	Item #	Checklist item	Location where item is reported
		used in the process.	
Effect measures	12	Specify for each outcome the effect measure(s) (e.g. risk ratio, mean difference) used in the synthesis or presentation of results.	4
Synthesis methods	13a	Describe the processes used to decide which studies were eligible for each synthesis (e.g. tabulating the study intervention characteristics and comparing against the planned groups for each synthesis (item #5)).	3, 4
	13b	Describe any methods required to prepare the data for presentation or synthesis, such as handling of missing summary statistics, or data conversions.	3, 4
	13c	Describe any methods used to tabulate or visually display results of individual studies and syntheses.	3, 4, Figure 1
	13d	Describe any methods used to synthesize results and provide a rationale for the choice(s). If meta-analysis was performed, describe the model(s), method(s) to identify the presence and extent of statistical heterogeneity, and software package(s) used.	-
	13e	Describe any methods used to explore possible causes of heterogeneity among study results (e.g. subgroup analysis, meta-regression).	-
	13f	Describe any sensitivity analyses conducted to assess robustness of the synthesized results.	-
Reporting bias assessment	14	Describe any methods used to assess risk of bias due to missing results in a synthesis (arising from reporting biases).	4
Certainty assessment	15	Describe any methods used to assess certainty (or confidence) in the body of evidence for an outcome.	4
**RESULTS**			
Study selection	16a	Describe the results of the search and selection process, from the number of records identified in the search to the number of studies included in the review, ideally using a flow diagram.	5, Figure 1
	16b	Cite studies that might appear to meet the inclusion criteria, but which were excluded, and explain why they were excluded.	Table S2
Study characteristics	17	Cite each included study and present its characteristics.	5-7, Table 1

PRISMA

PRISMA 2020 Checklist

Section and Topic	Item #	Checklist item	Location where item is reported
Risk of bias in studies	18	Present assessments of risk of bias for each included study.	8, Figure 2
Results of individual studies	19	For all outcomes, present, for each study: (a) summary statistics for each group (where appropriate) and (b) an effect estimate and its precision (e.g. confidence/credible interval), ideally using structured tables or plots.	-
Results of syntheses	20a	For each synthesis, briefly summarise the characteristics and risk of bias among contributing studies.	8
	20b	Present results of all statistical syntheses conducted. If meta-analysis was done, present for each the summary estimate and its precision (e.g. confidence/credible interval) and measures of statistical heterogeneity. If comparing groups, describe the direction of the effect.	-
	20c	Present results of all investigations of possible causes of heterogeneity among study results.	8-9
	20d	Present results of all sensitivity analyses conducted to assess the robustness of the synthesized results.	-
Reporting biases	21	Present assessments of risk of bias due to missing results (arising from reporting biases) for each synthesis assessed.	8
Certainty of evidence	22	Present assessments of certainty (or confidence) in the body of evidence for each outcome assessed.	8-9
**DISCUSSION**			
Discussion	23a	Provide a general interpretation of the results in the context of other evidence.	9-10
	23b	Discuss any limitations of the evidence included in the review.	9-10
	23c	Discuss any limitations of the review processes used.	9-10
	23d	Discuss implications of the results for practice, policy, and future research.	9-10
**OTHER INFORMATION**			
Registration and protocol	24a	Provide registration information for the review, including register name and registration number, or state that the review was not registered.	3
	24b	Indicate where the review protocol can be accessed, or state that a protocol was not prepared.	3
	24c	Describe and explain any amendments to information provided at registration or in the protocol.	-
Support	25	Describe sources of financial or non-financial support for the review, and the role of the funders or sponsors in the	11

Section and Topic	Item #	Checklist item	Location where item is reported
		review.	
Competing interests	26	Declare any competing interests of review authors.	11
Availability of data, code and other materials	27	Report which of the following are publicly available and where they can be found: template data collection forms; data extracted from included studies; data used for all analyses; analytic code; any other materials used in the review.	11

From: Page MJ, McKenzie JE, Bossuyt PM, Boutron I, Hoffmann TC, Mulrow CD, et al. The PRISMA 2020 statement: an updated guideline for reporting systematic reviews. BMJ 2021;372:n71. doi: 10.1136/bmj.n71. This work is licensed under CC BY 4.0. To view a copy of this license, visit https://creativecommons.org/licenses/by/4.0/
